# The clinical role of combined circulating complement C1q and AIP for CAD with LDL-C level below 1.8mmol/L

**DOI:** 10.1186/s12944-024-02127-8

**Published:** 2024-05-04

**Authors:** Chenyujun Hu, Zehao Zhao, Shutong Dong, Qianyun Guo, Yujie Zhou

**Affiliations:** Department of Cardiology, Beijing Anzhen Hospital, Beijing Institute of Heart Lung and Blood Vessel Disease, Beijing Key Laboratory of Precision Medicine of Coronary Atherosclerotic Disease, Clinical center for coronary heart disease, Capital Medical University, Capital Medical University, Beijing, 100029 China

**Keywords:** C1q, Atherogenic index of plasma, Coronary artery disease, Diabetes, LDL-C < 1.8mmol/L

## Abstract

**Background:**

In the past few years, circulating complement C1q involvement in atherosclerosis has garnered growing research interest in addition to the emerging recognition of the novel lipid marker named atherogenic index of plasma (AIP). Nevertheless, among patients experiencing low-density lipoprotein cholesterol (LDL-C) levels less than 1.8mmol/L, the interplay between C1q combined with the AIP for coronary artery disease (CAD) is ambiguous.

**Methods:**

Patients were stratified into a non-CAD and CAD group according to their coronary angiography. The association between C1q in conjunction with the AIP and CAD was explored using restricted cubic spline analyses and logistic regression models. To assess how it predicted, a receiver operating characteristic analysis was undertaken.

**Results:**

A total of 7270 patients comprised 1476 non-CAD patients and 5794 patients diagnosed with CAD were analyzed. A comparison of the two groups showed that the C1q levels were notably higher compared to the CAD group, while AIP exhibited an inverse trend. Across quartiles of C1q, the AIP demonstrated a decline with increasing C1q levels, and significant differences were observed between the groups. A correlation analysis underscored a notable negative correlation between the two variables. Univariate and multivariate logistic regression analyses revealed significant associations between CAD and the C1q quartile groups/AIP. Furthermore, compared with the Q4 group, a decrease in the C1q levels corresponded to an escalation in CAD risk, with the odds ratio rising from 1.661 to 2.314.

**Conclusions:**

In conclusion, there appears to be a notable positive correlation between the combination of C1q with the AIP and CAD.

**Supplementary Information:**

The online version contains supplementary material available at 10.1186/s12944-024-02127-8.

## Introduction

Atherosclerosis is the central pathological process that underlies coronary artery disease (CAD). It consists of inflammation, abnormal lipid levels, dysfunction of endothelial cells, and the growth along with movement of smooth muscle cells (SMC) within blood vessels [1]. Circulating complement C1q functions as a recognition element within the innate immune complement system, exhibiting a dual function in atherosclerosis. In the context of atherosclerotic plaques, C1q identifies anti-oxidized low-density lipoprotein autoantibodies or interacts directly with modified lipoproteins, consequently initiating the classical complement pathway [2, 3]. The potent innate immune reaction results in the production of inflammatory complement activation byproducts and the formation of membrane attack complexes. These processes advance disease development in animal models and are associated with the likelihood of atherosclerosis and cardiovascular ailments in humans [4, 5]. Nevertheless, macrophages have the capability to release C1q, indicating its existence within lesions during the initial stages of atherosclerosis, possibly even in the absence of other complement elements [6]. Circulating complement C1q is detected in regions afflicted with atherosclerosis, inflammation, and vascular lesions, underscoring the noteworthy involvement of this complement in vascular ailments. Nevertheless, its precise significance in these contexts remains uncertain [7]. The atherogenic index of plasma (AIP), computed using an equation lg [triglyceride (TG)/ high-density lipoprotein cholesterol (HDL-C)], has surfaced as a novel lipid indicator strongly linked to atherosclerosis [8]. Prior researches have confirmed the close link between AIP and CAD and its prognosis [9, 10]. Recent studies have recognized the AIP as a vital prognostic factor for unfavorable results among patients experiencing acute coronary syndromes (ACS) that then underwent percutaneous coronary intervention (PCI) while maintaining low-density lipoprotein cholesterol (LDL-C) levels beneath 1.8mmol/L [11]. Nonetheless, investigations pertaining to C1q and the AIP, which are closely associated with atherosclerosis, within populations exhibiting LDL-C levels less than 1.8mmol/L are lacking. The objective of this study is to investigate the clinical significance for circulating complement C1q and the AIP in combination for individuals diagnosed with CAD.

## Methods

### Study participants and study design

**Fig. 1:**
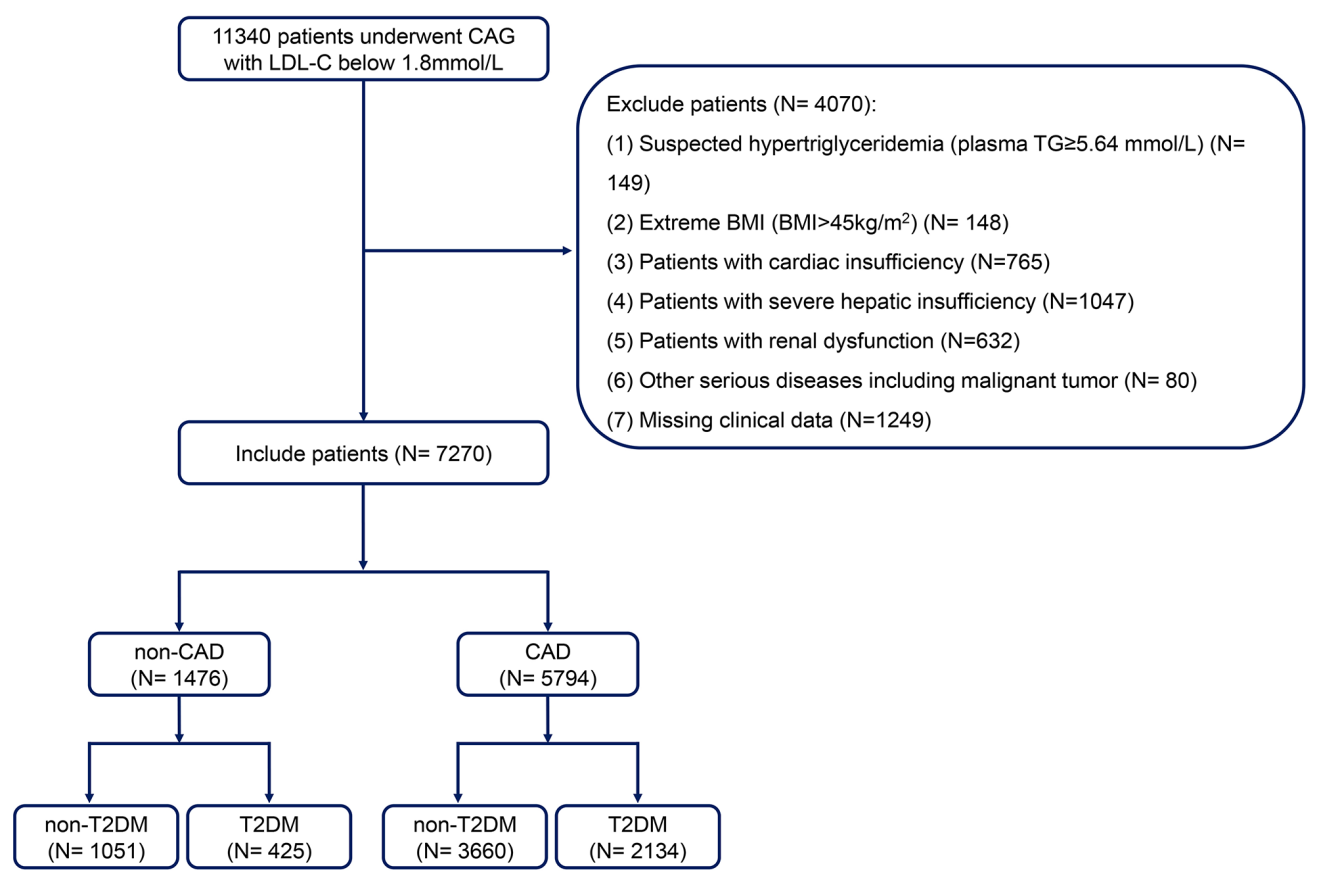
Study population enrollment CAG, coronary arteriography; LDL-C, low-density lipoprotein cholesterol; CAD, coronary artery disease; T2DM, type 2 diabetes mellitus

11,340 patients who underwent coronary arteriography between January 1, 2017 and January 1, 2019 were admitted to the Beijing Anzhen Hospital that was affiliated with the Capital Medical University. The exclusion conditions included: (1) patients with suspected hypertriglyceridemia in [plasma TG ≥ 5.64 mmol/L] (*N* = 149); (2) extreme body mass indexes (BMI) (BMI > 45 kg/m^2^) (*N* = 148); (3) patients with cardiac insufficiency (*N* = 765); (4) patients with severe hepatic insufficiency (*N* = 1047); (5) patients with renal dysfunction (*N* = 632); (6) other serious diseases including malignant tumors (*N* = 80) and (7) missing clinical data (*N* = 1249). Ultimately, 4070 patients were excluded. In agreement with the established criteria for diagnosing CAD, 7270 subjects were stratified into CAD (*n* = 5794) and non-CAD (*n* = 1476) groups (Fig. 1). All participants have signed informed consent forms.

### Data source and collection

In this study, all participants had venous blood drawn 12 h after fasting upon admission. General data included height, sex (male%), diastolic blood pressure (DBP), age, systolic blood pressure (SBP), weight, hypertension history, drinking history, hyperlipidemia history, type 2 diabetes mellitus (T2DM) history, smoking history, and family history of cardiovascular diseases (CVD). In addition, the fundamental drug therapies for diseases were the following: for CAD, aspirin, P2Y12 inhibitors (clopidogrel/ticagrelor), β-blockers, statins, and nitrates; for diabetic patients, oral antidiabetic drugs and insulin; for hyperlipidemia, statins and for hypertension, including β-blockers and angiotensin receptor blockers/angiotensin converting enzyme inhibitors (ARB/ACEI). The fundamental drug therapy was the same as that used for the overall population in the diabetic subgroup.

### Definitions

According to previous studies, the count of diseased coronary arteries was determined by tallying the main coronary arteries stenosed over 50%. Conversely, the control group exhibited no typical angina symptoms, with stenosis of the main coronary artery measuring less than 50% [10, 12]. The BMI was computed by using weight divided by the square of the height, and the AIP was calculated as lg (TG /HDL-C) [9].

### Measurement of the biochemical parameters

Biochemical indicators including the C-reactive protein (CRP), HDL-C, fasting blood glucose (FBG), LDL-C, aspartate aminotransferase (AST), total cholesterol (TC), TG, alanine aminotransferase (ALT), the estimated glomerular filtration rate (eGFR), creatinine (Cr), and glycated hemoglobin A1c (HbA1c), were acquired from the Hospital’s Department of Laboratory Medicine in accordance with established protocols. The C1q values were analyzed using an automatic biochemical analyzer (Hitachi-7600, Tokyo, Japan) according to the operational procedures and quality control criteria recommended in the instrument’s manual [13]. The left ventricular ejection fraction (LVEF) was assessed using a two-dimensional modified Simpson’s method via ultrasonic cardiography equipment.

### Statistical analysis

The study participants’ baseline characteristics were depicted using either the mean (SD) or median (interquartile range [IQR]), according to a continuous distribution of variables. The categorical variables were represented using the count (proportion). Categorical variables underwent analysis employing the chi-square test, while for the non-normally distributed data, the Kruskal-Wallis test was utilized. As for the normally distributed data, an analysis of variance (ANOVA) was used. For the non-normally distributed data, the correlation between two variables was evaluated utilizing a Spearman test. A Pearson correlation for the data with a normal distribution. In both the univariate and multivariate analyses, the relationship between C1q and the AIP with CAD was assessed using logistic regression models, indicating the 95% confidence interval (CI) and odds ratio (OR). To examine the impact of C1q in conjunction with the AIP on CAD as a continuous variable, restricted cubic spline (RCS) analysis with five knots was utilized. To assess the additional predictive value of C1q and the AIP beyond traditional risk factors in the baseline risk model that included age, sex, BMI, T2DM, and history of smoking, a receiver operator characteristic (ROC) curve analysis was conducted. DeLong’s test was used to compare the area beneath the curve (AUC) among the models. The statistical analyses were performed employing SPSS 22.0 and R 4.3.1, with a significance threshold of *P* < 0.05 established for all analyses.

## Results

### Clinical demographic features of the non-CAD and CAD groups

In total, 7270 participants (mean age: 60.81 ± 9.49 years; 77.1% men) were included in the current investigation (Table 1). A total of 1476 were categorized into the non-CAD group, while 5794 belonged to the CAD group. No notable distinctions were noted between both cohorts concerning age, BMI, SBP, DBP, history of hypertension, drinking history, history of family CVD, FBG, eGFR, and CRP. However, within the CAD group, the proportion of males, history of hyperlipidemia, smoking history, history of T2DM, and the levels of TC, TG, LDL-C, AIP, ALT, AST, HbA1c, and Cr were significantly higher in contrast to individuals within the non-CAD group. Conversely, the HDL-C, C1q, and LVEF levels demonstrated a significant decrease in the CAD group. Among the diabetic population, there were 2134 individuals with CAD and 425 individuals without CAD (Supplementary Table 1). It can be seen that the AIP levels within the CAD group exhibited significantly higher values compared to the non-CAD group, while the C1q levels were significantly lower.

**Table 1:** Baseline clinical and biochemical characteristics of the study population

Variables	Total (*n* = 7270)	non-CAD (*n* = 1476)	CAD (*n* = 5794)	*P* value
Age, year	60.81 ± 9.49	60.97 ± 9.51	60.77 ± 9.49	0.478
Sex, male%	5603 (77.1)	940 (63.7)	4663 (80.5)	< 0.001
BMI, kg/m^2^	25.43 ± 2.79	25.33 ± 2.87	25.46 ± 2.77	0.214
SBP, mmHg	127.88 ± 15.83	128.40 ± 15.22	127.73 ± 15.98	0.217
DBP, mmHg	74.95 ± 10.21	75.30 ± 9.64	74.86 ± 10.35	0.208
Hypertension, %	4579 (63.0)	921 (62.4)	3658 (63.1)	0.622
Hyperlipidemia, %	3990 (54.9)	773 (52.4)	3217 (55.5)	0.032
Smoking, %	3297 (45.4)	513 (34.8)	2784 (48.0)	< 0.001
Drinking, %	2006 (27.6)	384 (26.0)	1622 (28.0)	0.137
Family CVD, %	683 (9.4)	154 (10.4)	529 (9.1)	0.138
T2DM, %	2559 (35.2)	425 (28.8)	2134 (36.8)	< 0.001
TC, mmol/L	3.02 [2.75, 3.29]	3.00 [2.74, 3.27]	3.09 [2.81, 3.38]	< 0.001
TG, mmol/L	1.12 [0.80, 1.60]	1.07 [0.78, 1.53]	1.12 [0.81, 1.62]	0.011
HDL-C, mmol/L	1.03 [0.88, 1.21]	1.09 [0.92, 1.28]	1.01 [0.87, 1.19]	< 0.001
LDL -C, mmol/L	1.51 [1.32, 1.66]	1.51 [1.31, 1.66]	1.53 [1.34, 1.67]	0.004
AIP	1.03 [0.85, 1.23]	1.00 [0.81, 1.19]	1.04 [0.86, 1.24]	< 0.001
ALT, U/L	22.00 [16.00, 29.00]	21.00 [15.00, 28.00]	22.00 [16.00, 29.00]	< 0.001
AST, U/L	21.00 [18.00, 25.00]	20.00 [17.00, 25.00]	21.00 [18.00, 25.00]	0.002
FBG, mmol/L	6.00 [5.22, 7.80]	5.94 [5.22, 7.62]	6.01 [5.22, 7.83]	0.226
HbA1c, %	6.10 [5.70, 6.90]	5.90 [5.60, 6.60]	6.10 [5.70, 7.00]	< 0.001
eGFR, mL/min/1.73m^2^	97.35 [90.50, 104.03]	97.49 [90.45, 104.22]	97.30 [90.52, 104.00]	0.889
Cr, umol/L	69.10 [60.32, 78.30]	66.60 [57.30, 76.90]	69.50 [61.00, 78.60]	< 0.001
CRP, mg/L	3.50 [0.94, 13.12]	3.50 [0.90, 12.03]	3.92 [1.19, 18.06]	0.550
C1q, mg/L	162.40 [145.00, 179.50]	170.50 [153.07, 191.30]	160.50 [143.60, 177.07]	< 0.001
LVEF, %	65.00 [61.00, 68.00]	65.00 [62.00, 68.00]	65.00 [60.00, 68.00]	< 0.001
Aspirin, %	6587 (90.6)	1065 (72.2)	5522 (95.3)	< 0.001
P2Y12 inhibitors, %	4736 (65.1)	96 (6.5)	4640 (80.1)	< 0.001
Statins, %	6526 (89.8)	1139 (77.2)	5387 (93.0)	< 0.001
Nitrate, %	4172 (57.4)	428 (29.0)	3744 (64.6)	< 0.001
β-blockers, %	3900 (53.6)	598 (40.5)	3302 (57.0)	< 0.001
Insulin, %	566 (7.8)	99 (6.7)	467 (8.1)	0.094
Oral hypoglycemic drugs, %	1201 (16.5)	195 (13.2)	1006 (17.4)	< 0.001
ARB/ACEI, %	1258 (17.3)	236 (16.0)	1022 (17.6)	0.145

CAD, coronary artery disease; BMI, body mass index; SBP, systolic blood pressure; DBP, diastolic blood pressure; CVD, cardiovascular diseases; T2DM, type 2 diabetes mellitus; TC, total cholesterol; TG, triglyceride; HDL-C, high-density lipoprotein cholesterol; LDL-C, low-density lipoprotein cholesterol; AIP, atherogenic index of plasma; ALT, alanine aminotransferase; AST, aspartate aminotransferase; FBG, fasting blood glucose; HbA1c, glycated hemoglobin A1c; eGFR, estimated glomerular filtration rate; Cr, creatinine; CRP, C-reactive protein; LVEF, left ventricular ejection fraction; ARB, angiotensin receptor blockers; ACEI, angiotensin converting enzyme inhibitors

### Clinical demographic features of the four quartiles divided by the C1q levels

Based on the quartile distribution of C1q (1st quartile: 145.0, median: 162.4, 3rd quartile: 179.5), the study categorized patients into four groups: Q1, Q2, Q3, and Q4. Q1 had 1824 patients, Q2 had 1822 patients, Q3 had 1811 patients, and Q4 had 1813 patients (Table 2). No substantial variances were detected across the groups concerning the incidence of family CVD and hypertension, as well as levels of HDL-C, FBG, HbA1C, eGFR, and CRP. As the C1q levels increased, there was a gradual decrease in the proportion of males, smokers, drinkers, T2DM, and age. For the levels of the AIP, LDL-C, TG, TC, and Cr, and As for the C1q quartiles, the same monotone increasing trend was observed in the diabetic population (Supplementary Table 2).

**Table 2:** Baseline clinical and laboratory characteristics of the study patients according to the C1q quartiles

Variables	Q1 (*n* = 1824)	Q2 (*n* = 1822)	Q3 (*n* = 1811)	Q4 (*n* = 1813)	*P* value
Age, year	62.07 ± 9.37	60.97 ± 9.34	60.16 ± 9.52	60.02 ± 9.62	< 0.001
Sex, male%	1599 (87.7)	1471 (80.7)	1380 (76.2)	1153 (63.6)	< 0.001
BMI, kg/m^2^	25.54 ± 2.73	25.52 ± 2.82	25.46 ± 2.82	25.21 ± 2.79	0.012
SBP, mmHg	128.86 ± 15.69	128.08 ± 15.92	127.95 ± 15.65	126.56 ± 15.98	0.002
DBP, mmHg	75.85 ± 10.24	75.23 ± 10.31	74.87 ± 10.07	73.86 ± 10.13	< 0.001
Hypertension, %	1185 (65.0)	1143 (62.7)	1132 (62.5)	1119 (61.7)	0.208
Hyperlipidemia, %	1081 (59.3)	996 (54.7)	968 (53.5)	945 (52.1)	< 0.001
Smoking, %	899 (49.3)	865 (47.5)	827 (45.7)	706 (38.9)	< 0.001
Drinking, %	575 (31.5)	511 (28.0)	466 (25.7)	454 (25.0)	< 0.001
Family CVD, %	165 (9.0)	190 (10.4)	170 (9.4)	158 (8.7)	0.317
T2DM, %	690 (37.8)	628 (34.5)	641 (35.4)	600 (33.1)	0.023
TC, mmol/L	3.10 [2.85, 3.38]	3.04 [2.77, 3.33]	3.00 [2.72, 3.25]	2.93 [2.67, 3.20]	< 0.001
TG, mmol/L	1.26 [0.90, 1.87]	1.16 [0.84, 1.71]	1.08 [0.79, 1.53]	0.97 [0.71, 1.33]	< 0.001
HDL-C, mmol/L	1.02 [0.87, 1.22]	1.02 [0.88, 1.20]	1.03 [0.87, 1.21]	1.04 [0.88, 1.21]	0.304
LDL -C, mmol/L	1.55 [1.36, 1.68]	1.51 [1.32, 1.66]	1.51 [1.32, 1.67]	1.47 [1.26, 1.63]	< 0.001
AIP	1.09 [0.90, 1.30]	1.06 [0.87, 1.26]	1.02 [0.85, 1.21]	0.97 [0.80, 1.15]	< 0.001
ALT, U/L	22.00 [16.00, 30.00]	22.00 [17.00, 29.00]	21.00 [16.00, 28.00]	21.00 [16.00, 27.00]	< 0.001
AST, U/L	21.00 [18.00, 26.00]	21.00 [18.00, 26.00]	21.00 [18.00, 25.00]	21.00 [17.00, 24.00]	< 0.001
FBG, mmol/L	6.01 [5.20, 7.78]	6.01 [5.22, 7.83]	6.05 [5.26, 7.82]	5.93 [5.20, 7.77]	0.468
HbA1c, %	6.10 [5.70, 6.80]	6.10 [5.60, 6.90]	6.10 [5.60, 7.00]	6.10 [5.60, 6.90]	0.272
eGFR, mL/min/1.73m^2^	96.95 [89.71, 104.28]	97.33 [90.58, 103.59]	97.28 [90.45, 104.37]	97.83 [90.98, 104.14]	0.356
Cr, umol/L	70.40 [62.20, 79.20]	69.80 [61.50, 78.60]	68.60 [60.10, 77.50]	66.70 [57.40, 77.70]	< 0.001
CRP, mg/L	4.30 [2.08, 14.34]	3.23 [1.15, 8.72]	2.96 [0.78, 18.06]	2.14 [0.48, 17.58]	0.365
C1q, mg/L	133.90 [125.30, 139.60]	154.30 [150.00, 158.50]	170.60 [166.10, 175.00]	196.30 [187.70, 209.80]	< 0.001
LVEF, %	65.00 [61.00, 68.00]	65.00 [60.00, 67.00]	65.00 [60.00, 68.00]	65.00 [61.00, 68.00]	0.009
Aspirin, %	1652 (90.6)	1674 (91.9)	1647 (90.9)	1614 (89.0)	0.029
P2Y12 inhibitors, %	1260 (69.1)	1216 (66.7)	1175 (64.9)	1085 (59.8)	< 0.001
Statins, %	1656 (90.8)	1651 (90.6)	1609 (88.8)	1610 (88.8)	0.072
Nitrate, %	1092 (59.9)	1075 (59.0)	1059 (58.5)	946 (52.2)	< 0.001
β-blockers, %	976 (53.5)	966 (53.0)	1000 (55.2)	958 (52.8)	0.462
Insulin, %	123 (6.7)	154 (8.5)	154 (8.5)	135 (7.4)	0.140
Oral hypoglycemic drugs, %	333 (18.3)	298 (16.4)	302 (16.7)	268 (14.8)	0.045
ARB/ACEI, %	294 (16.1)	314 (17.2)	313 (17.3)	337 (18.6)	0.274

CAD, coronary artery disease; BMI, body mass index; SBP, systolic blood pressure; DBP, diastolic blood pressure; CVD, cardiovascular diseases; T2DM, type 2 diabetes mellitus; TC, total cholesterol; TG, triglyceride; HDL-C, high-density lipoprotein cholesterol; LDL-C, low-density lipoprotein cholesterol; AIP, atherogenic index of plasma; ALT, alanine aminotransferase; AST, aspartate aminotransferase; FBG, fasting blood glucose; HbA1c, glycated hemoglobin A1c; eGFR, estimated glomerular filtration rate; Cr, creatinine; CRP, C-reactive protein; LVEF, left ventricular ejection fraction; ARB, angiotensin receptor blockers; ACEI, angiotensin converting enzyme inhibitors

### Associations of the C1q and the AIP with CAD and other variables

The correlation analysis revealed a noteworthy negative correlation between C1q and CAD, while the AIP demonstrated a notable positive correlation with CAD. Moreover, C1q values displayed a significant negative correlation with AIP. Regarding other variables, C1q showed negative correlations with age, sex (male), BMI, SBP, DBP, hypertension history, smoking history, drinking history, T2DM, LDL-C, ALT, AST, and Cr, and positive correlations with eGFR. AIP exhibited negative correlations with the eGFR and positive correlations with age, sex (male), BMI, SBP, DBP, hypertension history, history of hyperlipidemia, smoking history, drinking history, T2DM, LDL-C, ALT, FBG, HbA1c, eGFR, and Cr (Table 3 and Supplementary Table 3).

**Table 3:** Correlation between C1q and AIP with other variables

Variables	C1q *R*	*P* value	AIP *R*	*P* value
Age	-0.081	< 0.001	0.187	< 0.001
Sex	-0.210	< 0.001	0.050	< 0.001
BMI	-0.030	0.038	0.298	< 0.001
SBP	-0.051	< 0.001	0.044	0.002
DBP	-0.065	< 0.001	0.040	< 0.001
Hypertension	-0.030	0.009	0.071	< 0.001
Hyperlipidemia	-0.0034	0.004	0.059	< 0.001
Smoking	-0.074	< 0.001	0.096	< 0.001
Drinking	-0.058	< 0.001	0.026	0.028
T2DM	-0.036	0.002	0.077	< 0.001
LDL-C	-0.097	< 0.001	0.047	< 0.001
AIP	-0.158	< 0.001	-	-
ALT	-0.046	0.001	0.134	< 0.001
AST	-0.060	< 0.001	0.023	0.105
FBG	-0.005	0.661	0.117	< 0.001
HbA1c	-0.017	0.189	0.113	< 0.001
eGFR	0.012	0.414	-0.055	< 0.001
Cr	-0.096	< 0.001	0.111	< 0.001
CRP	-0.113	0.097	0.029	0.673
C1q	-	-	-0.158	< 0.001
CAD	-0.152	< 0.001	0.063	< 0.001

AIP, atherogenic index of plasma; BMI, body mass index; SBP, systolic blood pressure; DBP, diastolic blood pressure; T2DM, type 2 diabetes mellitus; ALT, alanine aminotransferase; AST, aspartate aminotransferase; FBG, fasting blood glucose; HbA1c, glycated hemoglobin A1c; eGFR, estimated glomerular filtration rate; Cr, creatinine; CRP, C-reactive protein; CAD, coronary artery disease

### Predictive values of the C1q and the AIP in CAD

**Fig. 2:**
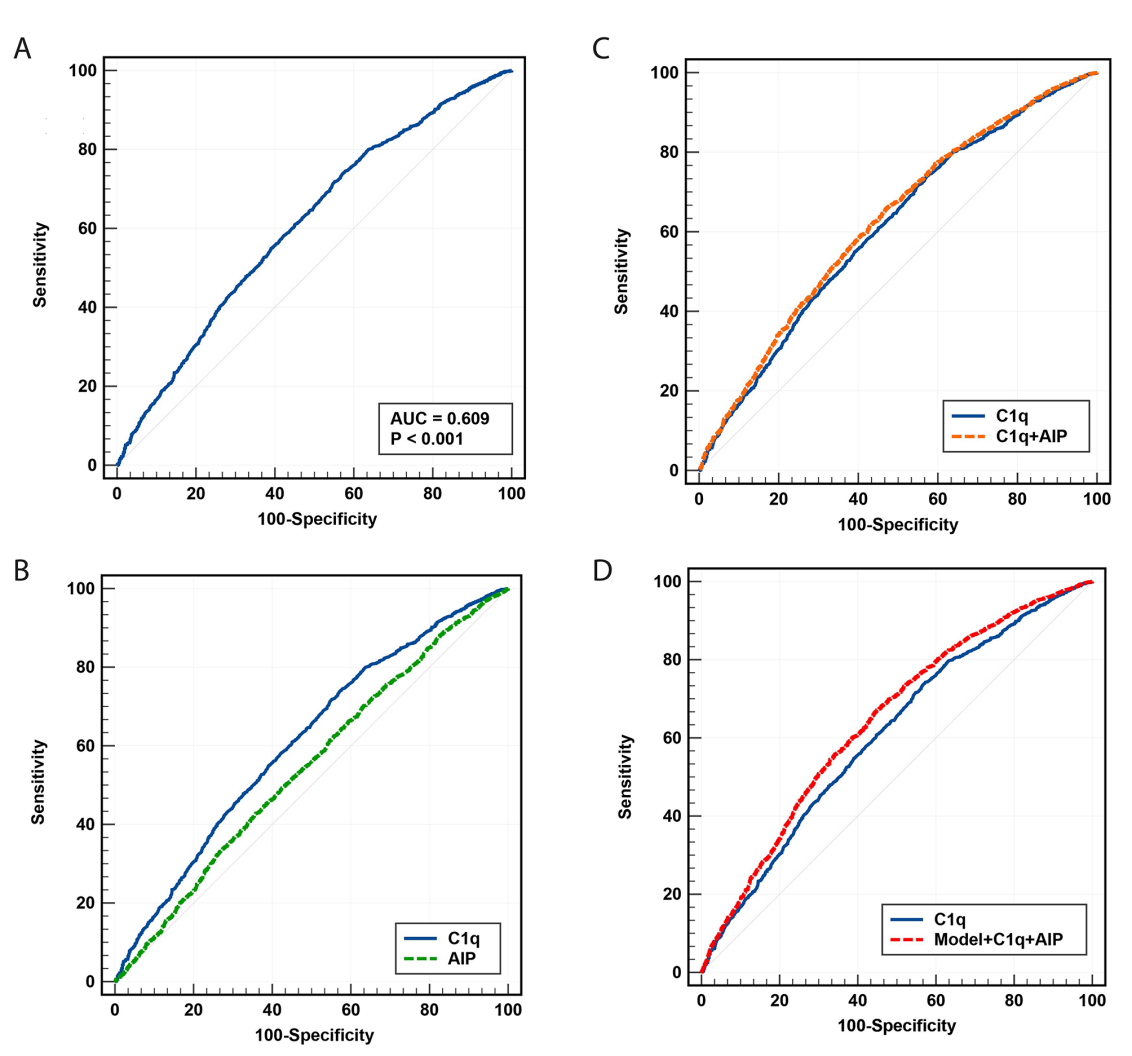
Receiver operator characteristic curve analysis (**A**) Predictive ability of C1q for CAD, (**B**) Comparison of predictive ability between C1q and AIP for CAD, (**C**) Comparison of predictive ability between C1q + AIP and C1q for CAD, (**D**) Comparison of predictive ability between C1q + AIP + Model and C1q for CAD. The model includes traditional risk factors which included age, sex, BMI, T2DM and history of smoking. AIP, atherogenic index of plasma

The predictive performance of C1q and the AIP was evaluated using a receiver operating characteristic curve analysis. As for predictive ability of C1q for CAD, the specificity was 42.68% and the sensitivity was 74.01% (Fig. 2A). In comparison to AIP (AUC = 0.545, 95% CI: 0.534–0.557, *P* < 0.001), the C1q levels (AUC = 0.609, 95% CI: 0.598–0.620, *P* < 0.001) demonstrated a superior predictive ability for the diagnosis (Fig. 2B). In addition, the combination of C1q and the AIP values yielded an enhanced performance (AUC = 0.624, 95% CI: 0.613–0.635, *P* < 0.001) compared with using either alone (Fig. 2C). The multivariate model that incorporated the AIP and C1q demonstrated improved discrimination (AUC = 0.646, 95% CI: 0.635–0.657, *P* < 0.001) (Fig. 2D). The predictive result for the diabetic group was shown in the Supplementary Fig. 1A-1D with the same trend as the entire population.

### Relationships between C1q with the variables and CAD

**Fig. 3:**
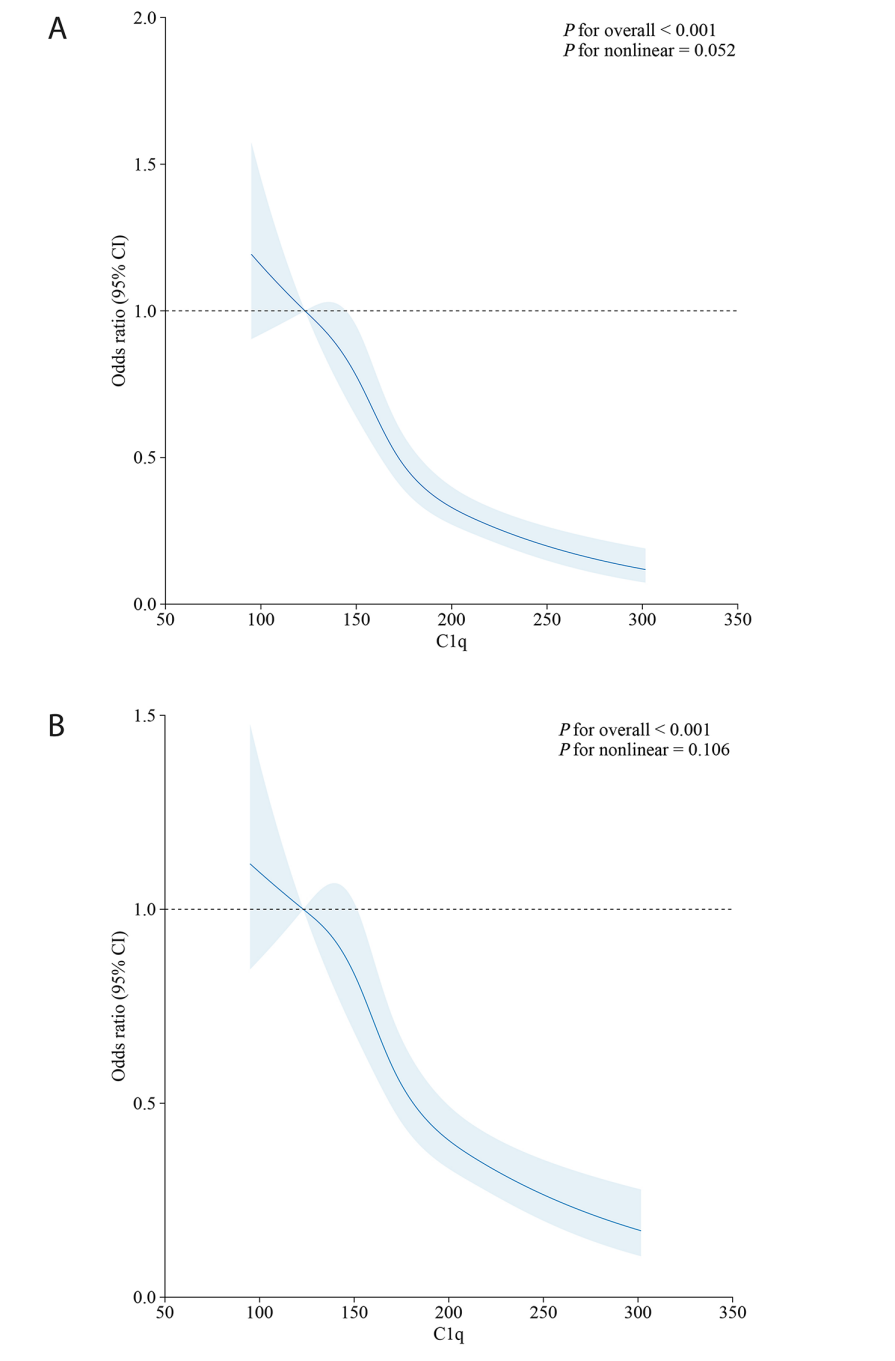
Association between C1q + AIP with or without the traditional factors and the prevalence of CAD (**A**) C1q combined with AIP for the prevalence of CAD without the traditional factors, (**B**) C1q combined with AIP for the prevalence of CAD with the traditional factors. The traditional risk factors included age, sex, BMI, T2DM and history of smoking. The ordinate represents the odds ratio value of coronary artery disease, while the abscissa represents the value of C1q

A notable positive correlation was observed in the univariate logistic regression analysis between sex (male), history of hyperlipidemia, T2DM history, smoking history, Cr, HbA1c, AIP, and CAD. In the logistic regression analysis with multi-variates, sex (male), smoking history, HbA1c, and the AIP showed significant positive correlations with CAD. With reference to Q4, as the C1q levels decreased from Q1 to Q3, the relationship with CAD showed progressively larger OR values in both univariate and multivariate regression analyses (Table 4). In the RCS curve, the combined presence of C1q and the AIP without (Fig. 3A) or with traditional risk factors (Fig. 3B) exhibited a consistent decreasing trend with CAD. The regression results with the same trend for the diabetic group were shown in Supplementary Tables 4 and Supplementary Fig. 2.

**Table 4:** Relationship between CAD and the C1q expressed as a categorical variable

Variable	Univariate analysis OR (95% CI)	*P* value	Multivariate analysis OR (95% CI)	*P* value
Age	0.998 (0.992–1.004)	0.477	1.003 (0.995–1.010)	0.496
Sex	2.351 (2.076–2.662)	< 0.001	1.754 (1.477–2.083)	< 0.001
Hypertension	1.032 (0.917–1.161)	0.601	0.914 (0.793–1.053)	0.914
Hyperlipidemia	1.135 (1.012–1.273)	0.013	1.067 (0.932–1.221)	0.346
T2DM	1.442 (1.273–1.633)	< 0.001	0.932 (0.773–1.124)	0.460
Smoking	1.736 (1.542–1.955)	< 0.001	1.285 (1.106–1.492)	0.001
Cr	1.105 (1.011–1.020)	< 0.001	1.001 (0.996–1.007)	0.642
HbA1c	1.227 (1.152–1.307)	< 0.001	1.260 (1.157–1.373)	< 0.001
AIP	1.709 (1.396–2.092)	< 0.001	1.750 (1.366–2.241)	< 0.001
CRP	1.010 (0.995–1.026)	0.202	1.026 (0.984–1.070)	0.220
C1q quartiles				
Q1	2.644 (2.243–3.117)	< 0.001	2.314 (1.901–2.816)	< 0.001
Q2	2.250 (1.919–2.638)	< 0.001	2.020 (1.678–2.431)	< 0.001
Q3	1.806 (1.550–2.104)	< 0.001	1.661 (1.390–1.986)	< 0.001
Q4	Reference		Reference	

T2DM, type 2 diabetes mellitus; Cr, creatinine; HbA1c, glycated hemoglobin A1c; AIP, atherogenic index of plasma; CRP, C-reactive protein

## Discussion

The present study first assessed the associations of the C1q and the AIP with CAD. The principal findings were the following: (1) Compared to the CAD group, the C1q levels displayed a marked elevation, whereas AIP demonstrated an inverse pattern. (2) Across quartiles of C1q, the AIP demonstrated a decline with increasing C1q levels, with significant differences observed between the groups. The correlation analysis underscored a notable negative correlation between the two variables. (3) The univariate and multivariate logistic regression analyses revealed significant associations between CAD and the C1q quartile groups/AIP. Furthermore, compared with the Q4 group, a decrease in the C1q levels corresponded to an escalation in the CAD risk, with the OR values rising from 1.661 to 2.314.

Research has indicated that the comparative levels of protein and mRNA expression of C1q exhibited a noteworthy increase in advanced atherosclerotic plaques compared with early lesions. Furthermore, the expression of C1q in the culprit plaques of patients diagnosed with ACS significantly surpassed that in patients with stable angina pectoris [14]. In recent clinical studies involving C1q, the serum complement C1q was significantly correlated with cardiovascular outcomes in ACS patients undergoing PCI treatment when levels of the highly-sensitive CRP (hs-CRP) were below 2 mg/L. This finding suggested the utility of C1q in stratifying the risk of ACS patients with reduced systemic inflammation [15]. In addition, studies have found that the activity of complement C1q was significantly higher in the CAD group [16]. This result contrasted with the results of some previous reports. Ni et al. found that the serum complement C1q levels, especially in ACS patients, particularly those with acute myocardial infarction (AMI), were lower. This suggested that further reduction in the complement C1q levels in ACS patients might be a precipitating factor for unstable or ruptured atherosclerotic plaques [17]. Similarly, Cavusoglu et al. found that in individuals with known or suspected CAD, decreased baseline plasma complement C1q levels were strong predictors of all-cause mortality and independent predictors of 10-year all-cause mortality [18]. In addition, Jia et al. found that compared with normal controls, circulating complement C1q levels were significantly reduced in CAD patients, while hs-CRP levels were significantly elevated. A multivariate logistic regression analysis revealed that decreased C1q and increased hs-CRP were independent risk factors for CAD [19]. Consistent with the latter findings, this study demonstrated that the levels of C1q were markedly elevated compared with those in the non-CAD group, and there was a notable inverse relationship observed between the C1q levels and the existence of CAD, including risk factors such as the AIP. However, the logistic regression analysis also suggested that decreased C1q was associated with an increased risk of CAD.

It has been speculated that the polarizing reasons for these studies may have been due to the dual role of the C1q in atherosclerosis and the complex role of C1q tumor necrosis factor-related protein (CTRP) in physiology and pathology. While it has been established that the activation of the complement by C1q can instigate inflammation and advance disease progression, intriguingly, in early-stage disease mouse models, C1q has exhibited a protective role against atherosclerosis [20]. In addition, investigations have revealed a complex relationship between C1q and the onset of CVD, suggesting that the initial stages of classical pathway activation may exert both protective and detrimental influences on human CVD development [21]. C1 inhibitors can inhibit neointimal plaque formation and inflammatory responses. This might entail the inhibition of complement activation, the suppression of leukocyte recruitment, and the lowering of triglyceride levels, thus providing a multi-modal approach to treating arterial diseases [22]. Prior research has illuminated that CTRP played a pivotal role in modulating numerous mechanisms on physiological and pathological, encompassing glucose and lipid homeostasis, cellular proliferation and apoptosis, protein kinase pathways, and inflammatory responses [23].

In recent years, increasing evidence suggested that the CTRP family plays multiple roles in endothelial function, glucose and lipid metabolism, inflammation regulation, and other processes. Consequently, the CTRP family exerted significant influences on various atherosclerotic cells, including the proliferation of vascular SMC, foam cell formation, and the dysfunction of endothelial cells [24]. Elevated levels of CTRP1 signified the extent and severity of atherosclerosis in human subjects, and it also had a causal role in atherosclerosis mice models both in vitro and in vivo [25]. CTRP3, CTRP9, and CTRP13 exhibited inhibitory effects on atherosclerosis [26]. Studies have found that CTRP3 overexpression can inhibit oxidative stress enhancement in myocardial infarction rat hearts. CTRP3 overexpression improved heart function and related myocardial fibrosis induced by myocardial infarction by inhibiting oxidative stress [27]. CTRP9 demonstrated significant protective effects against atherosclerosis, including enhancing plaque stability by activating AMPK, inhibiting levels of adhesion molecules in atherosclerotic plaques, and reducing MCP-1 and TNF-α secretion. Moreover, in ox-LDL-activated macrophages, by activating AMPK, CTRP9 downregulated the activity and expression of NLRP3, thus preventing atherosclerosis progression [28]. CTRP13 exerted protective effects in atherosclerosis by inhibiting macrophage inflammation and cholesterol metabolism [29].

C1q regulated the expression of genes associated with the toll-like receptor signaling, peroxisome proliferator-activated receptor signaling, and Janus kinase and signal transducer and activator of the transcription signaling pathway [30]. Moreover, the interaction between cholesterol crystals and complements may exert its effects by activating the NLRP3 inflammasome, thereby promoting the progression of atherosclerosis [31]. It was pointed out that zero prevention was the prevention of cardiovascular risk factors, including the goal of maintaining LDL-C levels under 1.8 mmol/L [32]. On real-world scenarios, individuals may experience ACS even with LDL-C levels under 1.8mmol/L. The question remained unanswered as to whether individuals with LDL-C levels under 1.8mmol/L, in addition no prior clinically established CVD should receive statin treatment [33]. Previous studies have also found that in this population, even when cholesterol has been reduced to a low level, there remained some more obvious risk factors [34]. Therefore, finding and identifying risk factors in this population was critical for their prognosis. As a novel lipid parameter in recent years, AIP has emerged as an outstanding biomarker, demonstrating remarkable value in numerous lipid-related studies. Furthermore, the relationship between inflammation and lipid profiles was inseparable. In this study, a significant negative correlation between C1q and the AIP was found. Moreover, when combining C1q and the AIP, they exhibited the maximum predictive value for CAD beyond the model of traditional risk factors. Considering the association between the AIP and diabetes, it has been found that T2DM involves multiple mechanisms in CAD, particularly insulin resistance and increased abnormalities in lipoproteins, and this promoted inflammatory vascular damage, thereby sustaining the process of atherosclerosis [35]. In addition, studies have indicated a J-shaped correlation between AIP and T2DM [36], and the AIP can predict the risk of CAD in T2DM patients [37]. Therefore, to explore whether T2DM affected the AIP levels, patients were also divided into a diabetic subgroup. An analysis of the subgroup data, showed that the overall trend in the diabetic subgroup was similar to the general population. Nonetheless, when considering AIP in this study, it was still necessary to consider the impact of T2DM on the AIP.

### Strengths and limitations

This study pioneered the exploration of associations between C1q, the AIP, and CAD, shedding light on potential biomarkers for this condition. In addition, this study collectively underscored the importance of integrating C1q and the AIP in the evaluation of CAD, offering valuable insights for clinical practice and future research endeavors. Moreover, this study highlighted the clinical implications, showing an escalation in the CAD risk with decreasing C1q levels, that could potentially guide risk assessments and management strategies. Furthermore, several limitations should be identified. First, it was a cross-sectional study lacking longitudinal data and patient follow-up. Second, regarding cardiovascular medication use, the study evaluated discharge prescriptions and reviewed medical histories, that may have not accurately reflected actual medication use. Third, indications and diagnoses related to interventions were not collected. Fourth, to assess whether the combination of C1q and AIP can serve as indicators of atherosclerosis progression and predictors of CVD, additional objective data were required, including intravascular imaging techniques and plaque pathology studies. The pathophysiological explanations presented in this study were speculative, and the underlying mechanisms remained unclear. Finally, some errors in the electronic health record system were commonly acknowledged issues in such cross-sectional studies. While data cleaning and standardization were conducted, confounding factors that could affect the robustness in this study may still exist.

## Conclusions

In summary, this study suggested a correlation between decreased C1q levels and CAD, and the combination of C1q and the AIP may have predictive value in CAD diagnosis. Moreover, the findings emphasized the clinical significance of integrating C1q and the AIP, which might potentially refine risk stratification models, guide personalized treatment approaches, and ultimately improve patient outcomes in CAD management. Therefore, the clinical relevance of this study offered tangible implications for enhancing the precision and effectiveness of CAD care, particularly in individuals with low LDL-C levels, where traditional risk assessment methods may fall short. Future longitudinal studies should explore the roles of C1q and AIP molecules in the clinical onset of CAD, tracking their expression levels across various phases to ascertain causality or correlation.

### Electronic supplementary material

Below is the link to the electronic supplementary material.

Supplementary Material 1

## Data Availability

No datasets were generated or analysed during the current study.

## Abbreviations

CAD: coronary artery disease
SMC: smooth muscle cells
AIP: atherogenic index of plasma
TG: triglyceride
HDL-C: high-density lipoprotein cholesterol
ACS: acute coronary syndromes
PCI: percutaneous coronary intervention
LDL-C: low-density lipoprotein cholesterol
BMI: body mass index
SBP: systolic blood pressure
DBP: diastolic blood pressure
T2DM: type 2 diabetes mellitus
CVD: cardiovascular diseases
TC: total cholesterol
AST: aspartate aminotransferase
ALT: alanine aminotransferase
FBG: fasting blood glucose
HbA1c: glycated hemoglobin A1c
eGFR: estimated glomerular filtration rate
Cr: creatinine
CRP: C-reactive protein
LVEF: left ventricular ejection fraction
OR: odds ratio
CI: confidence interval
RCS: restricted cubic spline
ROC: receiver operator characteristic
AUC: area under the curve
hs-CRP: high-sensitive C-reactive protein
CTRP: C1q tumor necrosis factor-related protein
