# Supplementary material for: The clinical role of combined circulating complement C1q and AIP for CAD with LDL-C level below 1.8mmol/L
Source: Lipids Health Dis. 2024 May 4;23:131. doi: 10.1186/s12944-024-02127-8 (PMC11070092; doi:10.1186/s12944-024-02127-8)
Supplement: Supplementary file 1 — Supplementary Material 1 [file 12944_2024_2127_MOESM1_ESM.docx]

**Table S1. Baseline clinical and biochemical characteristics of the diabetic population**

| Variables | Total (n=2559) | non-CAD (n=425) | CAD (n=2134) | *P* value |
| --- | --- | --- | --- | --- |
| Age, year | 61.53 ± 8.90 | 61.93 ± 8.68 | 61.45 ± 8.95 | 0.343 |
| Sex, male% | 1980 (77.4) | 273 (64.2) | 1707 (80.0) | <0.001 |
| BMI, kg/m^2^ | 25.71 ± 2.60 | 25.64 ± 2.60 | 26.08 ± 2.59 | 0.008 |
| SBP, mmHg | 128.77 ± 15.92 | 128.41 ± 16.01 | 130.58 ± 15.38 | 0.029 |
| DBP, mmHg | 74.05 ± 9.96 | 73.94 ± 10.12 | 74.56 ± 9.14 | 0.336 |
| Hypertension, % | 1775 (69.4) | 223 (52.5) | 1552 (72.7) | <0.001 |
| Hyperlipidemia, % | 1446 (56.5) | 243 (57.2) | 1203 (56.4) | 0.801 |
| Smoking, % | 1147 (44.8) | 150 (35.3) | 997 (46.7) | <0.001 |
| Drinking, % | 705 (27.5) | 122 (28.7) | 583 (27.3) | 0.600 |
| Family CVD, % | 239 (9.3) | 42 (9.9) | 197 (9.2) | 0.742 |
| TC, mmol/L | 2.97 [2.70, 3.25] | 2.95 [2.68, 3.23] | 3.03 [2.77, 3.35] | <0.001 |
| TG, mmol/L | 1.14 [0.83, 1.66] | 1.11 [0.82, 1.54] | 1.14 [0.84, 1.67] | 0.286 |
| HDL-C, mmol/L | 0.99 [0.85, 1.16] | 1.05 [0.90, 1.22] | 0.98 [0.84, 1.15] | <0.001 |
| LDL -C, mmol/L | 1.49 [1.30, 1.65] | 1.48 [1.29, 1.65] | 1.52 [1.34, 1.66] | 0.024 |
| AIP | 1.07 [0.88, 1.26] | 1.03 [0.84, 1.20] | 1.07 [0.89, 1.27] | 0.006 |
| ALT, U/L | 21.00 [16.00, 29.00] | 20.00 [15.00, 29.00] | 21.00 [16.00, 29.00] | 0.102 |
| AST, U/L | 20.00 [17.00, 24.00] | 20.00 [16.00, 25.00] | 20.00 [17.00, 24.00] | 0.541 |
| FBG, mmol/L | 7.77 [6.31, 10.22] | 7.80 [6.29, 10.42] | 7.77 [6.31, 10.22] | 0.767 |
| HbA1c, % | 7.20 [6.50, 8.10] | 7.00 [6.40, 7.90] | 7.20 [6.55, 8.20] | 0.003 |
| eGFR, mL/min/1.73m^2^ | 97.20 [89.75, 103.78] | 97.35 [89.11, 103.20] | 97.19 [89.90, 103.88] | 0.687 |
| Cr, umol/L | 68.40 [59.60, 77.95] | 67.50 [57.50, 76.90] | 68.55 [60.00, 78.27] | 0.017 |
| CRP, mg/L | 3.16 [0.90, 9.45] | 2.19 [0.85, 9.24] | 4.39 [1.44, 9.64] | 0.784 |
| C1q, mg/L | 161.50 [143.30, 178.50] | 168.10 [150.80, 187.50] | 159.50 [141.90, 176.90] | <0.001 |
| LVEF, % | 65.00 [60.00, 67.00] | 65.00 [62.00, 67.00] | 65.00 [60.00, 67.00] | 0.034 |
| Aspirin, % | 2375 (92.8) | 333 (78.4) | 2042 (95.7) | <0.001 |
| P2Y12 inhibitors, % | 1747 (68.3) | 30 (7.1) | 1717 (80.5) | <0.001 |
| Statins, % | 2352 (91.9) | 361 (84.9) | 1991 (93.3) | <0.001 |
| Nitrate, % | 1582 (61.8) | 152 (35.8) | 1430 (67.0) | <0.001 |
| β-blockers, % | 1467 (57.3) | 202 (47.5) | 1265 (59.3) | <0.001 |
| Insulin, % | 557 (21.8) | 99 (23.3) | 458 (21.5) | 0.440 |
| Oral hypoglycemic drugs, % | 1172 (45.8) | 190 (44.7) | 982 (46.0) | 0.658 |
| ARB/ACEI, % | 497 (19.4) | 85 (20.0) | 412 (19.3) | 0.793 |

CAD, coronary artery disease; BMI, body mass index; SBP, systolic blood pressure; DBP, diastolic blood pressure; CVD, cardiovascular diseases; TC, total cholesterol; TG, triglyceride; HDL-C, high-density lipoprotein cholesterol; LDL-C, low-density lipoprotein cholesterol; AIP, atherogenic index of plasma; ALT, alanine aminotransferase; AST, aspartate aminotransferase; FBG, fasting blood glucose; HbA1c, glycated hemoglobin A1c; eGFR, estimated glomerular filtration rate; Cr, creatinine; CRP, C-reactive protein; LVEF, left ventricular ejection fraction; ARB, angiotensin receptor blockers; ACEI, angiotensin converting enzyme inhibitors

**Table S2 Baseline clinical and laboratory characteristics of the diabetic patients according to the C1q quartiles.**

| Variables | Q1 (n=670) | Q2 (n=613) | Q3 (n=637) | Q4 (n=639) | *P* value |
| --- | --- | --- | --- | --- | --- |
| Age, year | 62.26 ± 8.94 | 62.03 ± 8.58 | 61.06 ± 8.91 | 60.76 ± 9.08 | 0.061 |
| Sex, male% | 598 (89.3) | 500 (81.6) | 496 (77.9) | 386 (60.4) | <0.001 |
| BMI, kg/m^2^ | 25.53 ± 2.59 | 25.95 ± 2.72 | 25.61 ± 2.50 | 25.78 ± 2.57 | 0.124 |
| SBP, mmHg | 130.57 ± 16.19 | 128.20 ± 15.14 | 128.99 ± 16.25 | 127.26 ± 15.86 | 0.070 |
| DBP, mmHg | 74.91 ± 9.84 | 74.33 ± 9.96 | 73.71 ± 10.16 | 73.24 ± 9.85 | 0.144 |
| Hypertension, % | 458 (68.4) | 446 (72.8) | 446 (70.0) | 425 (66.5) | 0.102 |
| Hyperlipidemia, % | 375 (56.0) | 318 (51.9) | 385 (60.4) | 368 (57.6) | 0.021 |
| Smoking, % | 333 (49.7) | 302 (49.3) | 278 (43.6) | 234 (36.6) | <0.001 |
| Drinking, % | 208 (31.0) | 188 (30.7) | 164 (25.7) | 145 (22.7) | 0.001 |
| Family CVD, % | 58 (8.7) | 67 (10.9) | 62 (9.7) | 52 (8.1) | 0.333 |
| TC, mmol/L | 3.07 [2.80, 3.34] | 2.99 [2.71, 3.26] | 2.96 [2.67, 3.23] | 2.88 [2.60, 3.13] | <0.001 |
| TG, mmol/L | 1.31 [0.94, 1.96] | 1.20 [0.87, 1.75] | 1.08 [0.80, 1.54] | 0.99 [0.75, 1.34] | <0.001 |
| HDL-C, mmol/L | 0.98 [0.84, 1.15] | 0.99 [0.84, 1.17] | 0.99 [0.85, 1.17] | 1.00 [0.86, 1.16] | 0.648 |
| LDL -C, mmol/L | 1.54 [1.35, 1.67] | 1.51 [1.30, 1.67] | 1.47 [1.31, 1.65] | 1.44 [1.25, 1.62] | <0.001 |
| AIP | 1.13 [0.94, 1.34] | 1.10 [0.90, 1.30] | 1.04 [0.87, 1.22] | 1.00 [0.83, 1.18] | <0.001 |
| ALT, U/L | 20.00 [16.00, 27.00] | 20.00 [16.00, 28.00] | 22.00 [16.00, 29.00] | 21.00 [16.00, 30.00] | 0.135 |
| AST, U/L | 20.00 [17.00, 24.00] | 20.00 [17.00, 24.00] | 20.00 [17.00, 25.00] | 20.00 [17.00, 25.00] | 0.131 |
| FBG, mmol/L | 7.98 [6.41, 10.22] | 7.88 [6.47, 10.64] | 7.78 [6.28, 10.18] | 7.42 [6.10, 9.99] | 0.009 |
| HbA1c, % | 7.40 [6.70, 8.30] | 7.30 [6.60, 8.20] | 7.10 [6.60, 8.00] | 6.90 [6.30, 7.70] | <0.001 |
| eGFR, mL/min/1.73m^2^ | 96.44 [88.16, 104.04] | 96.46 [90.11, 102.92] | 97.61 [90.51, 103.38] | 98.55 [90.83, 104.84] | 0.046 |
| Cr, umol/L | 69.60 [61.32, 78.00] | 69.50 [60.70, 78.60] | 68.10 [59.20, 77.80] | 66.20 [57.20, 77.20] | 0.001 |
| CRP, mg/L | 3.64 [2.51, 11.12] | 3.25 [0.78, 7.47] | 2.78 [1.02, 9.74] | 1.65 [0.76, 6.21] | 0.842 |
| C1q, mg/L | 132.60 [124.50, 138.00] | 153.30 [148.80, 157.50] | 169.60 [165.50, 174.00] | 194.90 [185.90, 206.70] | <0.001 |
| LVEF, % | 65.00 [60.00, 67.00] | 64.00 [60.00, 67.00] | 65.00 [60.00, 67.00] | 65.00 [61.00, 68.00] | 0.068 |
| Aspirin, % | 617 (92.1) | 566 (92.3) | 591 (92.8) | 601 (94.1) | 0.530 |
| P2Y12 inhibitors, % | 479 (71.5) | 414 (67.5) | 425 (66.7) | 429 (67.1) | 0.217 |
| Statins, % | 617 (92.1) | 565 (92.2) | 579 (90.9) | 591 (92.5) | 0.740 |
| Nitrate, % | 411 (61.3) | 380 (62.0) | 416 (65.3) | 375 (58.7) | 0.111 |
| β-blockers, % | 378 (56.4) | 350 (57.1) | 375 (58.9) | 364 (57.0) | 0.827 |
| Insulin, % | 121 (18.1) | 142 (23.2) | 149 (23.4) | 145 (22.7) | 0.060 |
| Oral hypoglycemic drugs, % | 318 (47.5) | 281 (45.8) | 289 (45.4) | 284 (44.4) | 0.737 |
| ARB/ACEI, % | 125 (18.7) | 127 (20.7) | 125 (19.6) | 120 (18.8) | 0.779 |

CAD, coronary artery disease; BMI, body mass index; SBP, systolic blood pressure; DBP, diastolic blood pressure; CVD, cardiovascular diseases; TC, total cholesterol; TG, triglyceride; HDL-C, high-density lipoprotein cholesterol; LDL-C, low-density lipoprotein cholesterol; AIP, atherogenic index of plasma; ALT, alanine aminotransferase; AST, aspartate aminotransferase; FBG, fasting blood glucose; HbA1c, glycated hemoglobin A1c; eGFR, estimated glomerular filtration rate; Cr, creatinine; CRP, C-reactive protein; LVEF, left ventricular ejection fraction; ARB, angiotensin receptor blockers; ACEI, angiotensin converting enzyme inhibitors

**Table S3. Correlation between C1q and AIP with other variables in diabetic patients**

| Variables | C1q  R | *P* value | AIP  R | *P* value |
| --- | --- | --- | --- | --- |
| Age | -0.083 | <0.001 | 0.192 | <0.001 |
| Sex (male) | -0.246 | <0.001 | 0.020 | 0.320 |
| BMI | -0.018 | 0.457 | 0.267 | <0.001 |
| SBP | -0.075 | 0.108 | 0.038 | 0.002 |
| DBP | -0.070 | 0.003 | 0.057 | 0.017 |
| Hypertension | -0.045 | 0.023 | 0.058 | 0.003 |
| Hyperlipidemia | -0.054 | 0.006 | 0.069 | <0.001 |
| Smoking | -0.090 | <0.001 | 0.092 | <0.001 |
| Drinking | -0.063 | 0.001 | 0.030 | 0.135 |
| LDL-C | -0.117 | <0.001 | 0.002 | 0.913 |
| AIP | -0.194 | <0.001 | - | - |
| ALT | -0.049 | 0.040 | 0.137 | <0.001 |
| AST | -0.048 | 0.041 | 0.029 | 0.227 |
| FBG | -0.071 | <0.001 | 0.086 | <0.001 |
| HbA1c | -0.143 | <0.001 | 0.111 | <0.001 |
| eGFR | 0.050 | <0.001 | -0.035 | 0.153 |
| Cr | -0.070 | <0.001 | 0.094 | <0.001 |
| CRP | -0.019 | 0.871 | 0.034 | 0.772 |
| C1q | - | - | -0.194 | <0.001 |
| CAD | -0.126 | <0.001 | 0.055 | 0.005 |

AIP, atherogenic index of plasma; BMI, body mass index; SBP, systolic blood pressure; DBP, diastolic blood pressure; ALT, alanine aminotransferase; AST, aspartate aminotransferase; FBG, fasting blood glucose; HbA1c, glycated hemoglobin A1c; eGFR, estimated glomerular filtration rate; Cr, creatinine; CRP, C-reactive protein; CAD, coronary artery disease.

**Table S4. Relationship between CAD and the C1q expressed as a categorical variable in diabetic patients**

| Variable | Univariate analysis  OR (95% CI) | *P* value | Multivariate analysis  OR (95% CI) | *P* value |
| --- | --- | --- | --- | --- |
| Age | 0.994 (0.982-1.006) | 0.312 | 1.003 (0.989-1.017) | 0.666 |
| Sex (male) | 2.226 (1.777-2.787) | <0.001 | 1.814 (1.340-2.455) | <0.001 |
| Hypertension | 1.072 (1.029-1.085) | 0.001 | 1.052 (1.006-1.081) | 0.036 |
| Hyperlipidemia | 0.968 (0.784-1.194) | 0.760 | 0.945 (0.746-1.197) | 0.640 |
| Smoking | 1.608 (1.295-1.996) | <0.001 | 1.191 (0.916-1.549) | 0.191 |
| Cr | 1.011 (1.003-1.018) | 0.007 | 0.999 (0.990-1.008) | 0.819 |
| HbA1c | 1.153 (1.046-1.270) | 0.004 | 1.192 (1.078-1.318) | 0.001 |
| AIP | 1.690 (1.168-2.446) | 0.005 | 1.979 (1.280-3.059) | 0.002 |
| CRP | 1.022 (0.980-1.065) | 0.318 | 1.021 (0.980-1.064) | 0.327 |
| C1q quartiles |  |  |  |  |
| Q1 | 2.369 (1.758-3.192) | <0.001 | 2.080 (1.470-2.944) | <0.001 |
| Q2 | 1.993 (1.485-2.675) | <0.001 | 1.944 (1.389-2.722) | <0.001 |
| Q3 | 1.463 (1.113-1.923) | <0.001 | 1.363 (1.006-1.866) | 0.035 |
| Q4 | Reference |  | Reference |  |

Cr, creatinine; HbA1c, glycated hemoglobin A1c; AIP, atherogenic index of plasma; CRP, C-reactive protein.

**Figure S1. Receiver operator characteristic curve analysis in diabetic patients**

**(A)
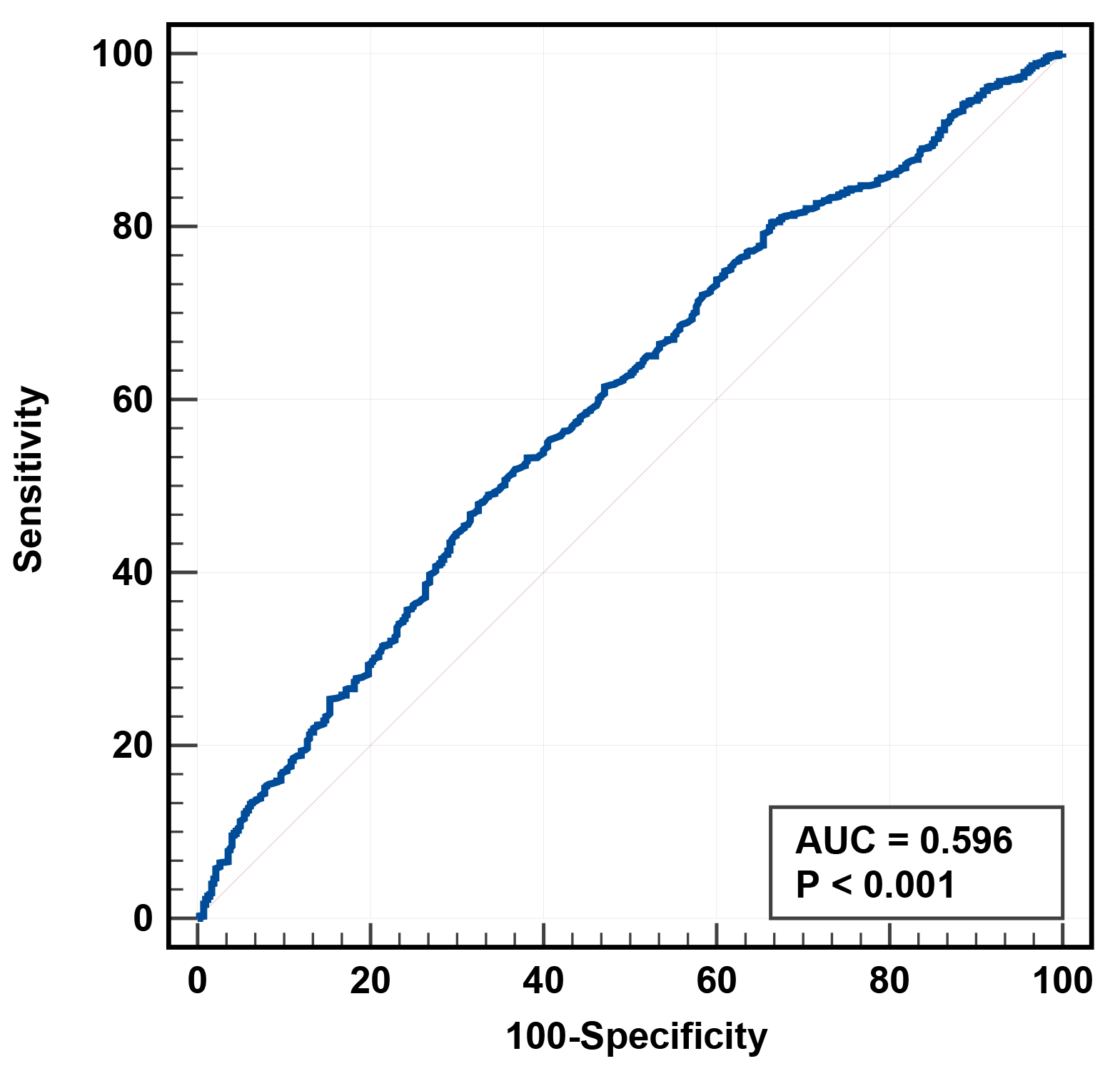
 (B)
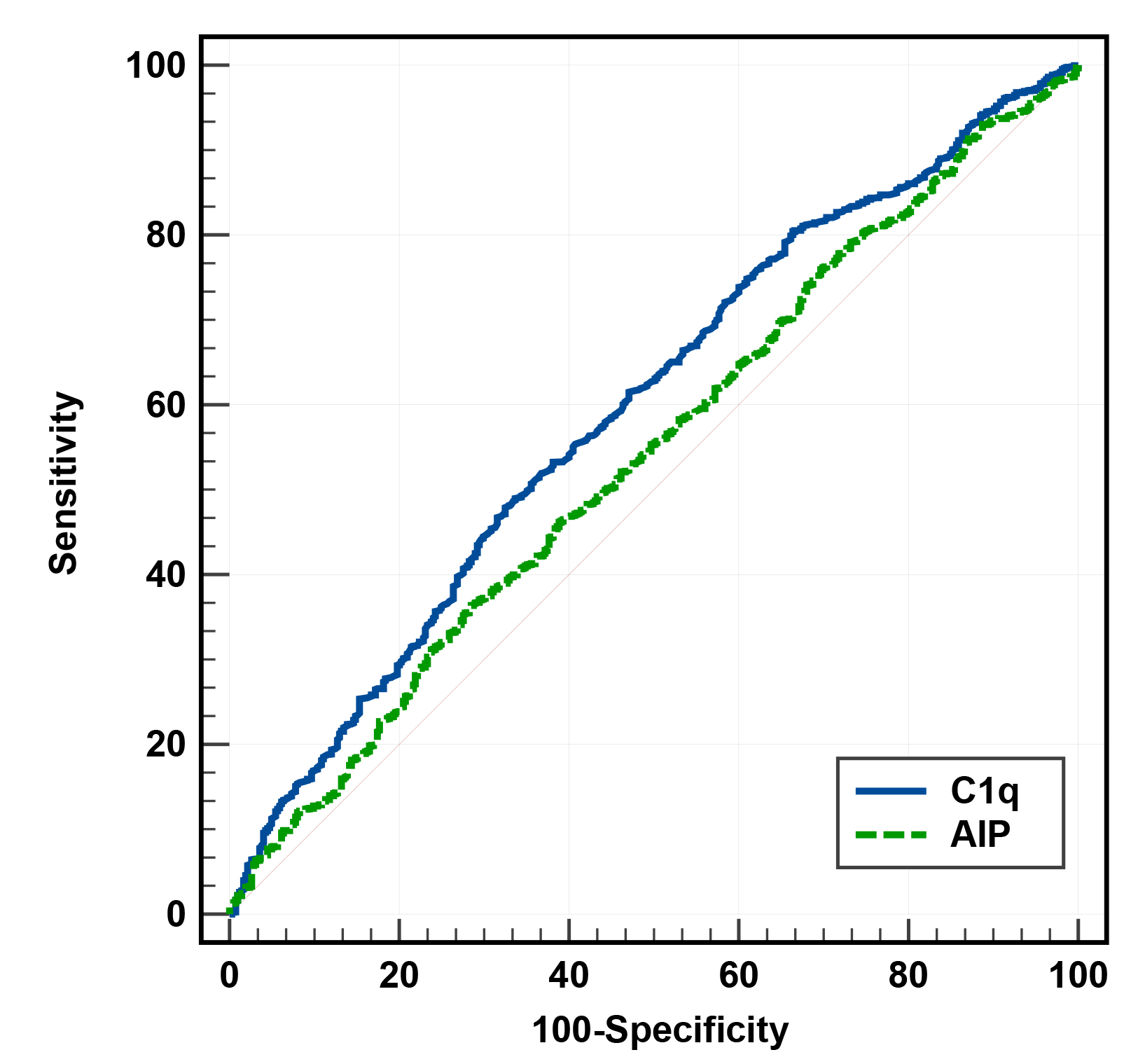
**

**(C)
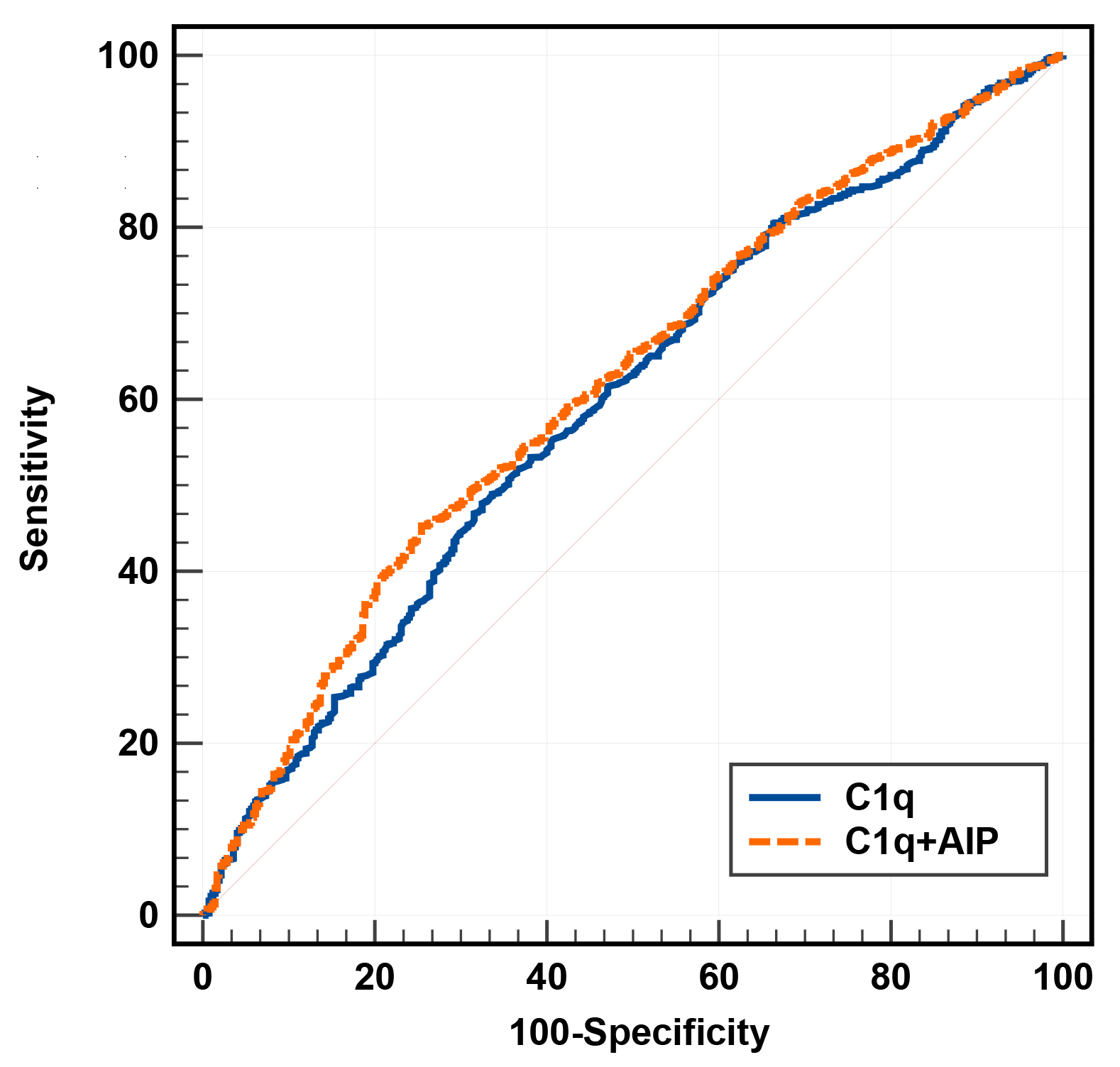
 (D)
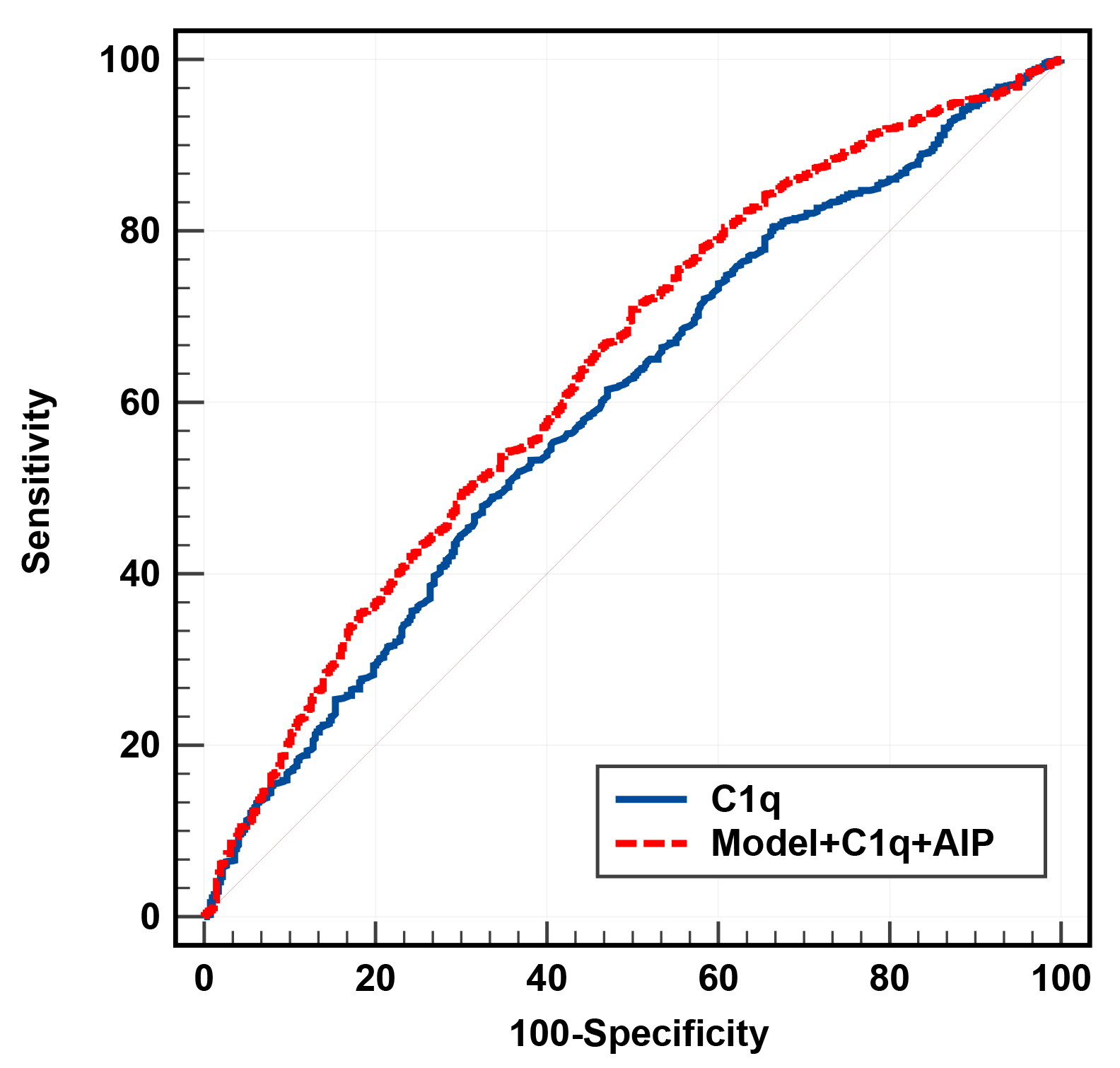
**

(A) Predictive ability of C1q for CAD, (B) Comparison of predictive ability between C1q and AIP for CAD, (C) Comparison of predictive ability between C1q+AIP and C1q for CAD, (D) Comparison of predictive ability between C1q+AIP+Model and C1q for CAD. The model includes traditional risk factors which included age, sex, BMI and history of smoking. AIP, atherogenic index of plasma.

**Figure S2. Association between C1q plus AIP with or without the traditional factors and the prevalence of CAD in diabetic patients**

**(A)**
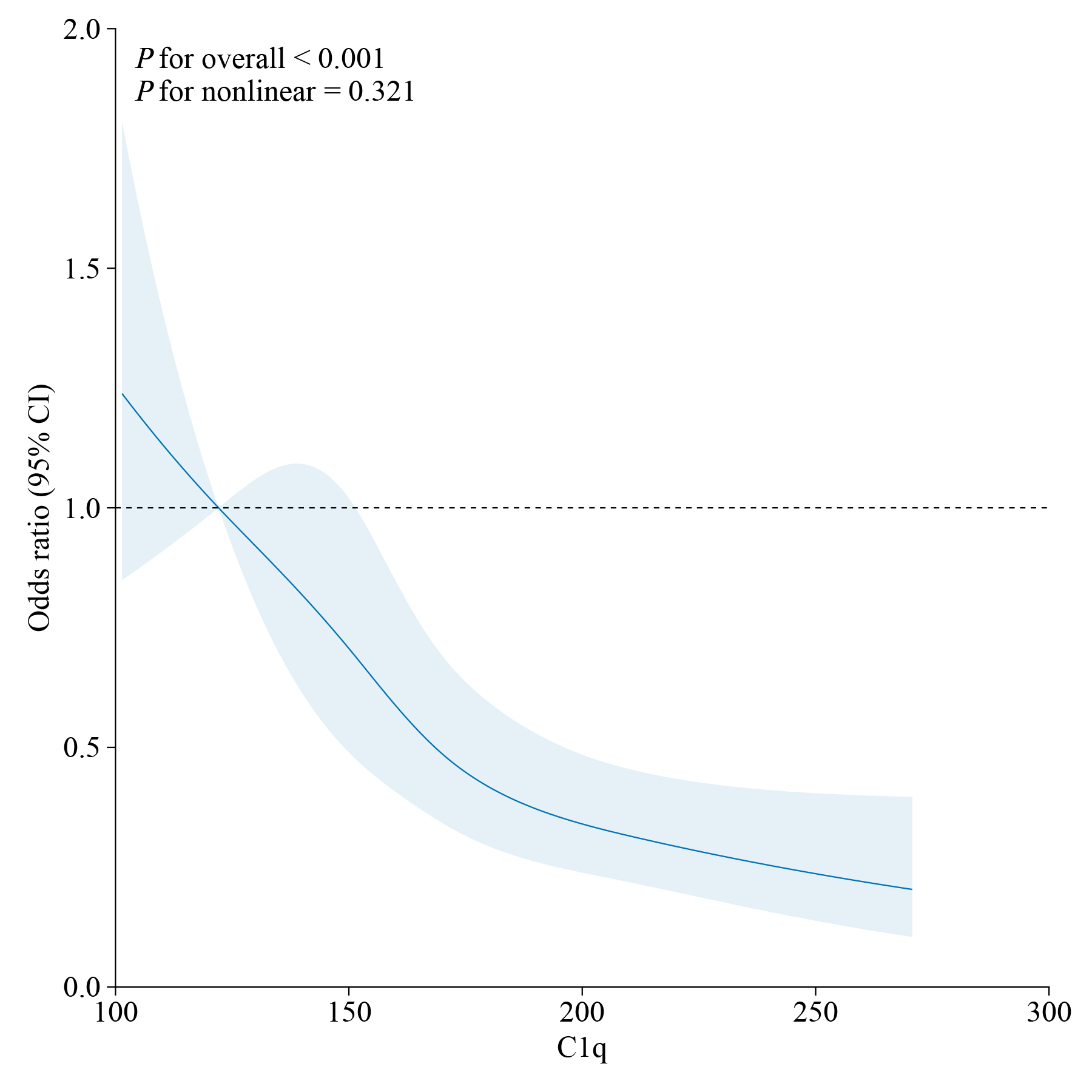
 **(B)**
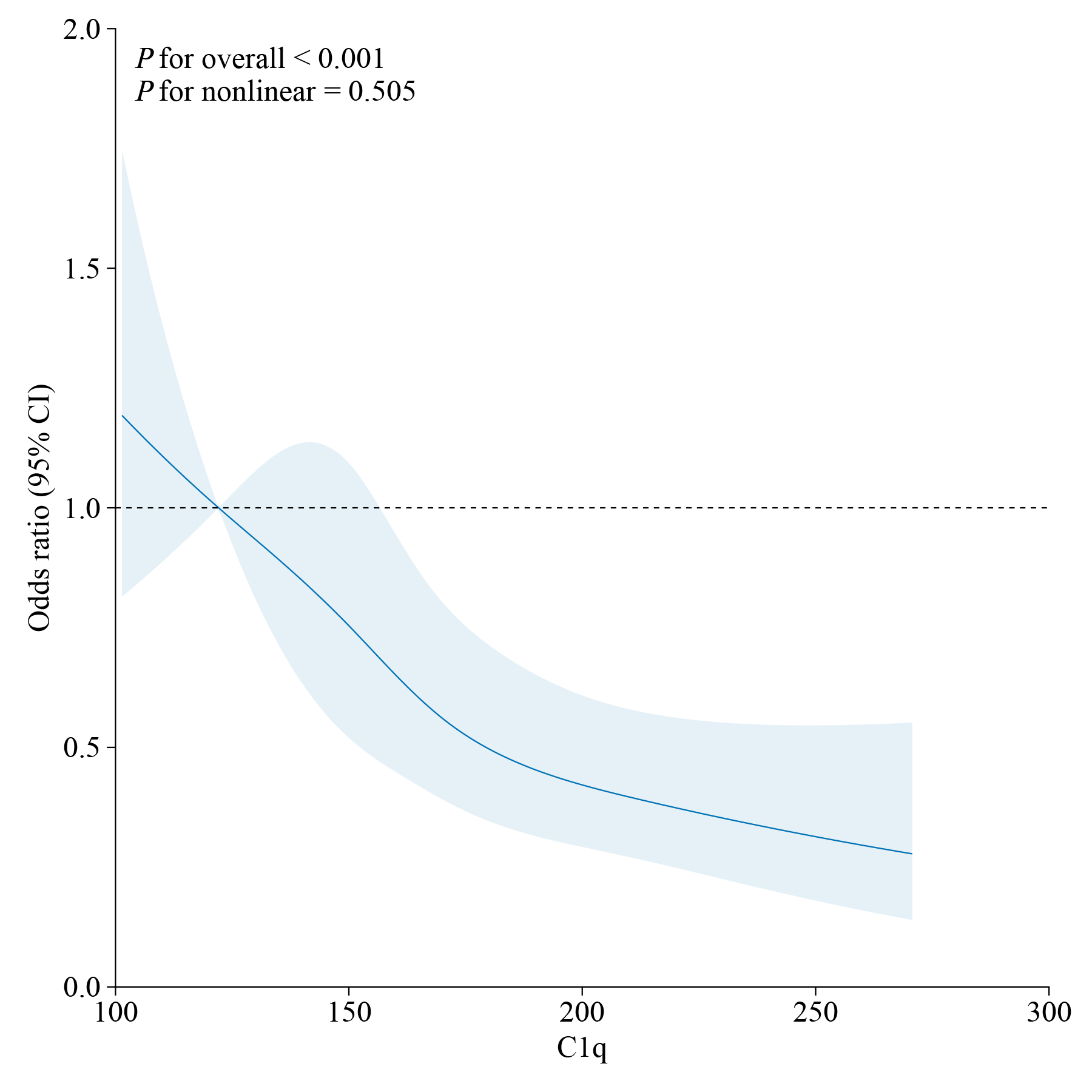


(A) C1q combined with AIP for the prevalence of CAD without the traditional factors, (B) C1q combined with AIP for the prevalence of CAD with the traditional factors. The traditional risk factors included age, sex, BMI and history of smoking. The ordinate represents the odds ratio value of coronary artery disease, while the abscissa represents the value of C1q.
